# Freezing of gait and fall detection in Parkinson’s disease using wearable sensors: a systematic review

**DOI:** 10.1007/s00415-017-8424-0

**Published:** 2017-03-01

**Authors:** Ana Lígia Silva de Lima, Luc J. W. Evers, Tim Hahn, Lauren Bataille, Jamie L. Hamilton, Max A. Little, Yasuyuki Okuma, Bastiaan R. Bloem, Marjan J. Faber

**Affiliations:** 1 Radboud university medical center, Donders Institute for Brain, Cognition and Behavior, Nijmegen, The Netherlands; 2 Department of Neurology, Radboud university medical center, Nijmegen, The Netherlands; 3 CAPES Foundation, Ministry of Education of Brazil, Brasília, DF Brazil; 4 Michael J Fox Foundation for Parkinson’s Research, New York, USA; 5 Aston University, Birmingham, UK; 6 Media Lab, Massachusetts Institute of Technology, Cambridge, USA; 7 Department of Neurology, Juntendo University Shizuoka Hospital, Izunokuni, Shizuoka Japan; 8 Radboud university medical center, Radboud Institute for Health Sciences, Scientific Center for Quality of Healthcare, Nijmegen, The Netherlands

**Keywords:** Parkinson’s disease, Ambulatory monitoring, Wearable sensors, Validation studies

## Abstract

Despite the large number of studies that have investigated the use of wearable sensors to detect gait disturbances such as Freezing of gait (FOG) and falls, there is little consensus regarding appropriate methodologies for how to optimally apply such devices. Here, an overview of the use of wearable systems to assess FOG and falls in Parkinson’s disease (PD) and validation performance is presented. A systematic search in the PubMed and Web of Science databases was performed using a group of concept key words. The final search was performed in January 2017, and articles were selected based upon a set of eligibility criteria. In total, 27 articles were selected. Of those, 23 related to FOG and 4 to falls. FOG studies were performed in either laboratory or home settings, with sample sizes ranging from 1 PD up to 48 PD presenting Hoehn and Yahr stage from 2 to 4. The shin was the most common sensor location and accelerometer was the most frequently used sensor type. Validity measures ranged from 73–100% for sensitivity and 67–100% for specificity. Falls and fall risk studies were all home-based, including samples sizes of 1 PD up to 107 PD, mostly using one sensor containing accelerometers, worn at various body locations. Despite the promising validation initiatives reported in these studies, they were all performed in relatively small sample sizes, and there was a significant variability in outcomes measured and results reported. Given these limitations, the validation of sensor-derived assessments of PD features would benefit from more focused research efforts, increased collaboration among researchers, aligning data collection protocols, and sharing data sets.

## Introduction

Parkinson’s disease (PD) is a progressive neurodegenerative disease characterized by four major motor signs: rest tremor, rigidity, bradykinesia, and postural instability [1]. Non-motor impairments, including executive dysfunctions, memory disturbances, and reduced ability to smell, are also seen in the disease [2–4]. Gait difficulties and balance issues are a disabling problem in many patients with PD, with different contributing factors, such as freezing of gait (FOG), festination, shuffling steps, and a progressive loss of postural reflexes. Its importance is underlined by a high prevalence of fall incidents in PD, especially in the later stages of the disease [5–7].

FOG is defined as a sudden and brief episode of inability to produce effective forward stepping [8]. The phenomenon is closely related to falls, appearing mainly during gait initiation, turning while performing a concomitant concurrent activity (i.e., dual tasks), or approaching narrow spaces [9–13]. Similar to FOG, fall episodes occur mainly during a half-turn or while dual tasking [6]. With disease progression, the increase of FOG and falling episodes, as well as the decrease in effectiveness of dopaminergic therapy amplify the burden related to these symptoms [6, 12, 14].

The management of gait disturbances, such as FOG and falls, often includes pharmacological interventions [12]. However, there is a growing interest in non-pharmacological interventions, such as physiotherapy [15], deep brain stimulation [16], or cueing devices [17, 18]. In all cases, reliable tools are required to determine the severity of gait disorders and evaluate the efficacy of interventions [5].

A number of subjective rating scales are used to evaluate motor symptoms, but most of them have limited validity and reliability [19]. To overcome these limitations, wearable sensors are emerging as new tools to objectively and continuously obtain information about patients’ motor symptoms [20–22]. These sensors, typically consisting of embedded accelerometers, gyroscopes and other, have been used to determine PD-related symptoms, including gait disorders [17, 18, 23–28]. They can act as an extension of health-professionals’ evaluation of PD symptoms, improving treatment, and augmenting self-management [29, 30].

Despite a large number of studies that investigated the use of wearable sensors to detect gait disturbances, such as FOG and falls, there is little agreement regarding the most effective system design, e.g., type of sensors, number of sensors, location of the sensors on the body, and signal processing algorithms. Here, we provide an overview of the use of wearable systems to assess FOG and falls in PD, with emphasis on device setup and results from validation procedures.

## Review methodology

A systematic search in the PubMed and Web of Science databases was performed in accordance with the PRISMA statement [31]. These databases were chosen to allow both medical and engineering journals to be included in the search process.

The search query, based on the PICO strategy [31], included Parkinson’s disease representing the Population, wearable, sensors, device representing the Intervention and falls or freezing of gait representing the Comparison. Outcome was not included as a key word to keep the query broad. The truncation symbol (*) and title/abstract filter were used to both broaden the search and provide more specificity. The final search query is shown in Table 1.

**Table 1:** Search queries used for each database

Database	Query	Hits
Web of science	(((TI = (sensor*) OR TS = (sensor*) OR TI = (device*) OR TS = (device*) OR TS = (wearable*) OR TI = (wearable*)) AND (TS = (freezing*) OR TI = (freezing*) OR TI = (fall*) OR TS = (fall*)) AND (TI = (Parkinson’s*) OR TS = (Parkinson’s*))))	272
PubMed	((“Freezing of gait” [tiab] OR Freezing* [tiab] OR fall* [tiab]) AND (wearable* [tiab] OR sensor* [tiab] OR device* [tiab]) AND Parkinson* [tiab])	280

The final search was performed in January 2017. In addition to the database search, a search in the references of review articles and book chapters that appeared during the search was performed. The goal was to identify potentially eligible articles absent in the database search.

Articles were selected based upon a set of eligibility criteria. As the objective of this review was to provide an overview of articles published on the topic, selection criteria were kept broad. Therefore, studies were included if they (1) present original research on the validation of wearable sensors (i.e., a single or combination of body worn computer/sensor [32, 33]) to detect, measure or monitor FOG, falls, or fall risk and (2) were performed in Parkinson’s disease patients. Studies were excluded if they (1) only used wearables to deliver cueing for FOG, (2) were published in languages other than English, or (3) did not provide sufficient information about study design and results.

Data extraction was performed using a predefined table. Variables extracted included: author, sample size, device usage (i.e., type of sensor, number of sensors, and location of the device), data collection procedures, and validation results. Validity was considered as the extent to which an instrument is measuring a concept that it is supposed to measure. It can be further divided into different types of validity, such as criterion-referenced validity, construct validity and content validity. In the case of wearable sensors, researchers are often interested in criterion-referenced validity, which can be assessed by the correlation between the sensor-derived outcome and the outcome of a reference instrument that has already been validated [34, 35]. Construct validity, also known as discriminant validity, is commonly used by assessing the extent to which groups that are supposed to produce different outcomes, indeed do so, for example, by comparing PD with non-PD, or DBS ON with DBS OFF.

## Results

### Selection process

**Fig. 1:**
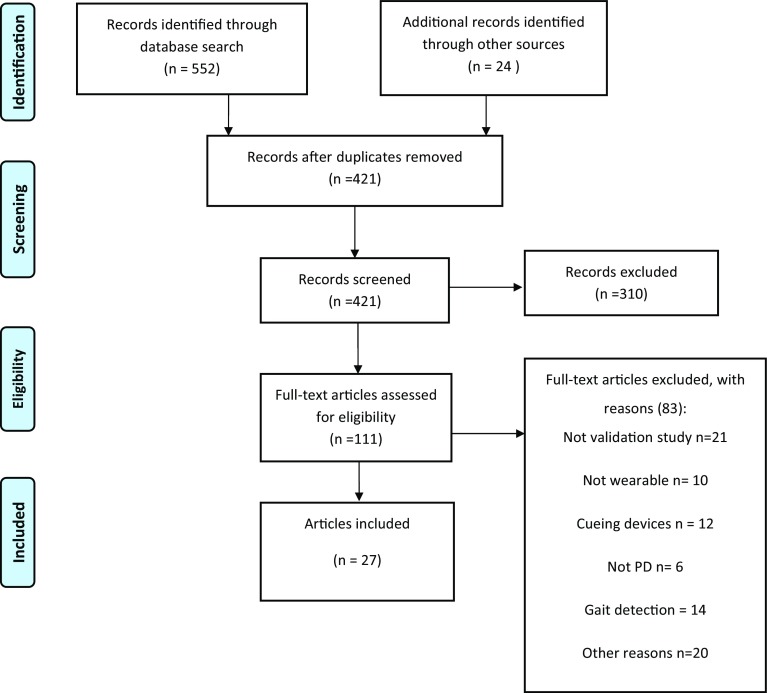
Selection process for eligible articles

In total, 552 articles were retrieved by the query. The selection process led to the final inclusion of 27 articles. Of those, 23 articles related to FOG, and 4 to falls. A complete overview of the selection process is presented in Fig. 1.

### Methodologies

#### FOG detection

A total of 23 articles investigated the use of wearable sensors to assess FOG in PD [18, 28, 36–56] (Table 2). The sample sizes varied from 1 [28] to 48 PD [51] per study, with a non-PD group being included in a few studies [28, 40, 48, 51, 53, 56]. Disease severity, when reported, ranged from 2 to 4 according to the Hoehn and Yahr scale. Data were collected according to three types of protocols: (1) a set of structured tasks performed in a laboratory environment (*n* = 18); (2) a protocol performed in a laboratory environment in which at least a part of which was designed to capture naturalistic behaviour (*n* = 2); and (3) natural or naturalistic behaviour in a home environment (*n* = 3).

**Table 2:** Characteristics of studies that investigated wearable sensors for FOG detection (*n* = 23)

Authors	Sample	Device locations (n)	Type of sensor	Procedures	ON	OFF	References	Validity results	Tested for cueing
FOG detection at home									
Martín [36]	6 PD FOG+	Waist (1)	Accelerometer	4 Different activities: (1) showing the home, (2) a FOG provocation test, (3) a short walk outdoors and (4) walking with a dual task activity. Also: a false positive protocol	✓	✓	Labeled video	Sensitivity: 91.7% Specificity: 87.4%	–
Ahlrichs [37]	8 PD FOG+ 12 PD FOG-	Waist (1)	Accelerometer	Scripted activities simulating natural behaviour at the patients’ homes	✓	✓	Labeled video	Sensitivity: 92.3% Specificity: 100%	–
Tzallas [38]	*Lab* 24 PD FOG unknown *Home* 12 PD FOG unknown	Wrist (2) Shin (2) Waist (1)	Accelerometer Gyroscope	*Lab* A series of tasks *Home* 5 consecutive days of free living	✓	✓	*Lab* Live annotation by clinician, confirmed by video analysis *Home* Self-reports (no further details provided)	*Lab* *Accuracy* 79% (sensitivity and specificity not reported) *Home* Mean absolute error: 0.79 (no further explanation provided; accuracy, sensitivity and specificity not reported)	–
FOG detection at the laboratory (“free” elements included in protocol)									
Mazilu [39]	5 PD FOG+	Shin (2)	Accelerometer Gyroscope Magnetometer	3 Sessions on 3 different days (2 consisting of walking tasks, 1 “free” walking in hospital and park)	?	?	Labeled video	Sensitivity: 97% Specificity: not reported (only reported: false positives count: 27 vs. 99 true positives)	✓
Cole [40]	10 PD FOG unknown 2 non-PD	Forearm ACC (1) Thigh ACC (1) Shin ACC & EMG (1)	Accelerometer EMG	Unscripted and unconstrained activities of daily living in apartment-like setting	?	?	Labeled video	Sensitivity: 82.9% Specificity: 97.3%	–
FOG detection at the laboratory (only tasks)									
Rezvanian [41]	Same as used in [17]	Shin (1) Thigh (1) Lower back (1)	Accelerometer	Same as used in [2]	✓	✓	Same as used in [2]	Sensitivity/specificity Shin only: 84.9/81% Thigh only: 73.6/79.6% Lower back only: 83.5/67.2%	–
Zach [42]	23 PD FOG+	Waist (1)	Accelerometer	A series of walking tasks	–	✓	Labeled video	Sensitivity: 78% Specificity: 76%	–
Kim [43]	15 PD FOG+	Waist (1) Trouser pocket (1) Shin (1)	Accelerometer Gyroscope	walking task (with single and dual tasking)	?	?	Labeled video.	Sensitivity/specificity Waist only: 86/92% Trouser pocket only: 84/92% Shin only: 81/91%	–
Coste [44]	4 PD FOG unknown	Shin (1)	Accelerometer Gyroscope Magnetometer	Walking task with dual tasking	?	?	Labeled video	Sensitivity: 79.5% Specificity: not reported (only number of falls positives: 13 vs. 35 true positives)	–
Kwon [45]	12 PD FOG+	Shoe (2)	Accelerometer	A walking task	✓	–	Labeled video	Sensitivity: 86% (from graph) Specificity: 86% (from graph)	–
Yungher [46]	14 PD FOG+	Lower back (1) Thigh (2) Shin (2) Feet (2)	Accelerometer Gyroscope Magnetometer	TUG in a 5-m course.	–	✓	Labeled video	No validity/reliability measures were reported	–
Djuric-Jovici [47]	12 PD FOG unknown	Shin (1)	Accelerometer Gyroscope	To walk along a complex pathway, created to provoke freezing episodes	–	✓	Labeled video	Sensitivity/specificity FOG with tremor: 100/99% FOG with complete motor block: 100/100%	–
Tripoliti [48]	11 PD FOG+ 5 non-PD	Wrist (2) Shin (2) Waist (1) Chest (1)	Accelerometer Gyroscope	A series of walking tasks	✓	✓	Live annotation by clinician, confirmed by video analysis	Sensitivity: 81.94% Specificity: 98.74%	–
Moore [49]	25 PD FOG+	Lower back (1) Thigh (2) Shin (2) Feet (2)	Accelerometer	TUG on a standardized 5-m course	–	✓	Labeled video	ICC number of FOG/ICC percent time frozen/sensitivity/specificity All sensors: 0.75/0.80/84.3/78.4% 1 shin only: 0.75/0.73/86.2/66.7% Lower back only: 0.63/0.49/86.8/82.4%	–
Morris [50]	10 PD FOG+	Shin (2)	Accelerometer	TUG on a standardized 5-m course	–	✓	Labeled video	ICC for number of FOG episodes: 0.78 ICC for percentage time frozen: 0.93	–
Mancini [51]	21 PD FOG+ 27 PD FOG- 21 non-PD	Lower back (1) Shin (2)	Accelerometer Gyroscope	3 Times the extended length iTUG	–	✓	FOG scale and ABC scale, and comparison between groups (PD FOG+, PD FOG- and non-PD)	*Criterion validity* Frequency ratio and FOG scale: *p* = 0.6, *p* = 0.002 Frequency Ratio and ABC scale: *p* = −0.47, *p* = 0.02 *Discriminant validity* Frequency ratio was larger in PD FOG+ compared to PD FOG- (*p* = 0.001), and in PD FOG- versus non-PD (*p* = 0.007)	–
Niazmand [52]	6 PD FOG+	Thigh (2) Shin (2) Bellybutton (1) (sensors embedded in pants)	Accelerometer	A series of walking tasks	?	?	Labeled video	Sensitivity: 88.3% Specificity: 85.3%	–
Bachlin [17]	10 PD FOG+	Shin (1)	Accelerometer	A series of walking tasks	✓	✓	Labeled video	Sensitivity: 73.1% Specificity: 81.6%	✓
Jovanov [28]	1 PD FOG unknown 4 non-PD	Knee (1)	Accelerometer Gyroscope	Walking task.	?	?	Labeled video	No validity measures were reported	✓
Moore [53]	11 PD FOG+ 10 non-PD	Shin (1)	Accelerometer	Walking task along complex pathway to provoke FOG	✓	✓	Labeled video	Sensitivity without calibration: 78% Sensitivity with calibration: 89% Specificity not reported	–
Mancini [56]	16 PD FOG+ 12 PD FOG-14 non-PD	Shin (2) Waist (1)	Accelerometer Gyroscope	TUG on a 7-m course Turning 360 in place for 2 min	–	✓	Labeled video	*Criterion validity* Freezing ratio duration × clinical ratings: *p* = 0.7, *p* = 0.003 Freezing Ratio duration × FOG questionnaire: *p* = 0.5, *p* = 0.03	–
Capecci [55]	20 PD FOG+	Waist (1)	Accelerometer	TUG on a standardized 5-m course	✓	–	Labeled video	Algorithm 1/Algorithm 2 Sensitivity: 70.2/87.5% Specificity: 84.1/94.9% Precision: 63.4/69.5% Accuracy: 81.6/84.3% AUC: 0.81/0.90	–
Handojoseno [54]	4 PD FOG+	Scalp (8)	EEG	TUG on a standardized 5-m course	✓	–	Labeled video	Sensitivity occipital channel: 74.6% Specificity occipital channel: 48.4% Accuracy occipital channel: 68.6%	–

*FOG* freezing of gait, *PD* Parkinson’s disease, *FOG+* PD patients with diagnosed freezing of gait events, *FOG*: PD patients with no diagnosed freezing of gait events, *SC* skin conductivity, *ECG* electrocardiogram, *non-PD* participants that have not been diagnosed with PDm *ACC* three tri-axial accelerometer, *TUG* timed-up-and-go test, *ICC* Intraclass correlation, *iTUG* automated timed-up-and-go test, *FOG questionnaire* freezing of gait questionnaire, *ABC scale* the activities-specific balance confidence scale, *AUC* area under curve

**Fig. 2:**
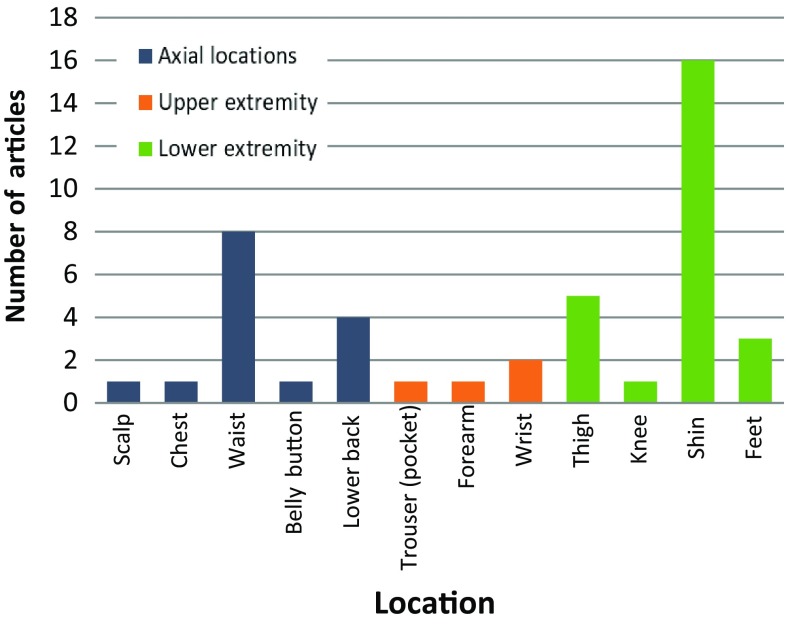
Distribution of device body location for FOG measurement

The types of sensors embedded in the devices worn by the participants varied. Tri-axial accelerometers were used in 22 articles, either as a single sensor (48%, *n* = 11), or combined with gyroscopes (35%, *n* = 8), or magnetometers (13%, *n* = 3). One study used electroencephalogram to measure changes in the brain activity from pre-determined areas during FOG episodes. Regarding the number of body locations, 56% (*n* = 13) of the studies utilized one location, while the other 44% (*n* = 10) used a combination of two or more locations. The shin (66% of studies, *n* = 16; 4 times used as the single location) and waist (33% of studies, *n* = 8; 3 times as the single location) were the most common body locations for the devices, although nine other locations were also explored (Fig. 2).

#### Falls: detection and fall risk analysis

Four articles on falls were retrieved: one article on fall detection and three articles presented the use of wearable sensors for analyzing fall risk. All protocols were performed in a home-based setting (Table 3) [57–60], and the sample size varied from one patient in a case report [57] up to 107 PD in a cross-sectional study [59]. One study reported disease severity and had an average Hoehn and Yahr score of 2.6 ± 0.7 [59]. All studies used tri-axial accelerometers. One study combined this sensor with force and bending sensors [58]; another with gyroscopes [60]. Sensor body locations included chest, insole (i.e., under the arch of the foot), and lower back.

**Table 3:** Characteristics of studies that investigated wearable sensors for fall and fall risk (*n* = 4)

Authors	Sample	Device location (*n*)	Type of sensor	Measure(s)	Procedures	ON	OFF	References	Validity results
Fall detection at home									
Tamura [57]	1 PD	Chest (1)	Accelerometer	Detection of falls	Participant carried the sensor in daily life	✓	✓	Fall diary	*Criterion validity* 19 out of 22 falls were detected. Specificity/false positives not reported
Fall risk at home									
Ayena [58]	7 PD 12 Young non-PD 10 Elderly non-PD	Insole (4)	Accelerometer Force sensor Bending sensor	Proposed new OLST score (with incorporation of both iOLST and score derived from balance model)	Participants performed the OLST at home as part of a serious game for balance training	✓	–	iOLST score Comparison between groups (PD vs young non-PD vs elderly non-PD, ground type)	*Criterion validity:* Proposed OLST score was not significantly different from iOLST score in all groups *Discriminant validity* - Proposed OLST score was significantly different between PD and non-PD subjects - Proposed OLST score was significantly differed between ground types
Weiss [59]	107 PD	Lower back (1)	Accelerometer	Anterior-posterior width of dominant frequency	Patients wore the sensor for 3 consecutive days at home	✓	✓	Comparison with BBT, DGI and TUG Among non-fallers: time until 1st fall during 1-year follow-up Comparison between fallers (*n* = 40) and non-fallers (*n* = 67) based on fall history	*Criterion validity* Anterior-posterior width was significantly correlated with BBT (r = -0.30), DGI (*r* = −0.25) and TUG (*r* = 0.32) Among non-fallers: anterior-posterior width significantly associated with time until 1st fall (*p* = 0.0039, Cox regression corrected for covariates) *Discriminant validity* Anterior-posterior width was larger (*p* = 0.012) in the fallers compared to the non-fallers
Iluz [60]	40 PD	Lower back (1)	Accelerometer Gyroscopes	Detection of missteps	*Laboratory* Walking tasks designed to provoke missteps (including dual tasking and negotiating with obstacles) *Home* Participants worn the devices for 3 days during day time	✓	✓	*Laboratory* Notation by clinicians Labeled video *Home* Comparison of groups (fallers vs. non-fallers)	*Criterion validity* Laboratory: Hit ratio: 93.1% Specificity: 98.6% *Discriminant validity* Home: Odds ratio of detection 1 or more missteps in fallers vs non-fallers: 1.84 (*p* = 0.010, 95% confidence interval 1.15–2.93)

*PD* Parkinson’s disease patients, *OLST* one-leg standing test, *iOLST* automatic one-leg standing test, *BBT* Berg balance test, *DGI* dynamic gait index, TUG, timed-up-and-go

### Validation

#### FOG detection

Among the 23 articles investigating FOG detection, 18 reported measures of validation performance (e.g., sensitivity, specificity, or accuracy) [17, 36–45, 47–49, 52–55], three studies used correlation measures, correlating the wearable-derived measure with the period of freezing or number of FOG events [50, 51, 56], and two studies did not report validity measures [28, 46].

**Fig. 3:**
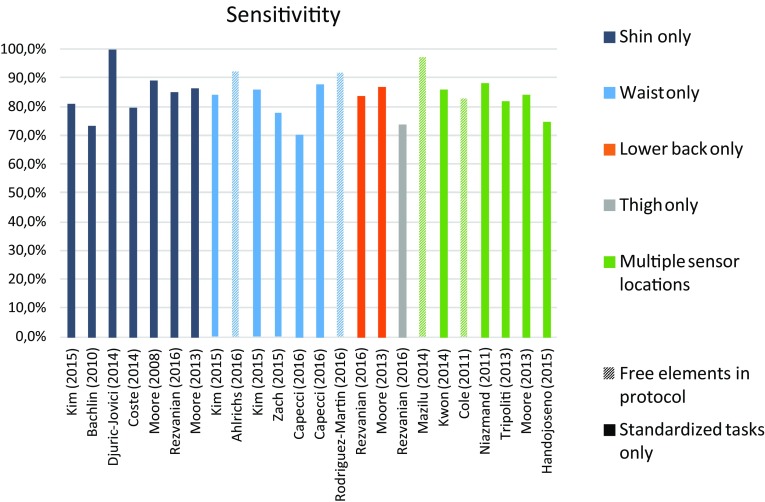
Instrument performance (sensitivity) in FOG detection

**Fig. 4:**
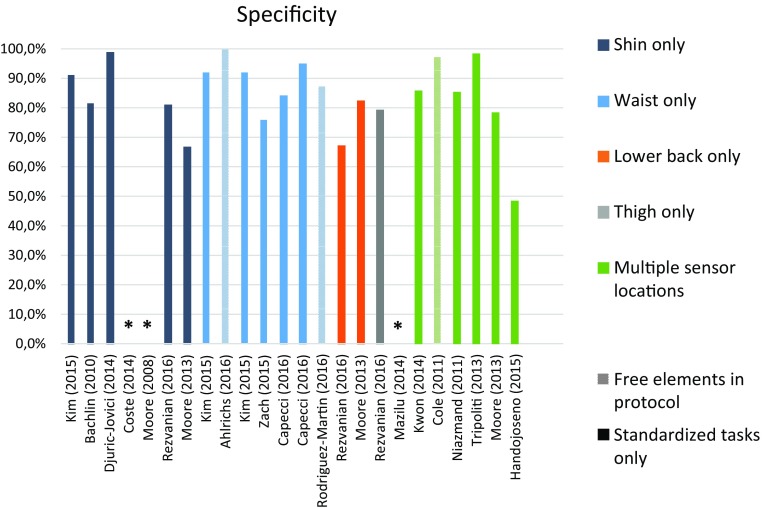
Instrument performance (specificity) in FOG detection. *Not reported

Overall, validity values ranged from 73 to 100% for sensitivity, and from 67 to 100% for specificity, and accuracy ranged from 68% up to 96%. Validity measures are summarized and compared across protocol setups in Figs. 3 and 4.

#### Fall detection and fall risk analysis

One article investigated the use of wearable sensors to detect falls, by comparing the data from a self-reported diary to the sensor data. The sensor captured 19 fall events from a total of 22 self-reported events [57].

Three articles presented the use of wearable sensors for analyzing fall risk. All of them reported discriminant validity by comparing sensor-derived outcomes between different groups, such as fallers and non-fallers or PD versus non-PD (see Table 3 for details). Weiss et al. [59] reported an illustrative approach, whereby the 107 participating PD patients wore one sensor in the lower back and made diary annotations about fall events. The sensor data, collected remotely in the patient’s home, were subsequently used to calculate a fall risk index. The time until first fall was significantly lower in subjects with a higher variable gait pattern (log rank test: *p* = 0.0018, Wilcoxon test: *p* = 0.0014).

## Discussion

This review included 27 articles, 23 on FOG, and four on falls. FOG studies were performed either in a laboratory or at home, with different types of protocols (structured versus free-movement). The shin (16/28 studies) was the most common device location and tri-axial accelerometers (26/28 studies) the most common sensor type. Sensitivity ranged from 73% to 100% and specificity ranged from 67% to 100% for the detection of FOG. Fall and fall risk studies were all home-based, using mostly one device (3/4 studies) containing tri-axial accelerometers. Sensors were positioned on the chest, insole, and lower back. The systems detected falls or quantified fall risk by various approaches and with varying degrees of validity.

### FOG detection

The results in this review support the potential for wearable devices. In the laboratory, systems showed a moderate to high specificity and sensitivity, which are in line with other evidence that wearable systems detecting FOG are already well validated in a laboratory setting [30]. Moreover, promising results were also achieved in studies performed in the home environment. Interestingly, the comparison of validity measures in terms of sensitivity and specificity (Figs. 3, 4) suggests that wearable sensors are able to accurately detect FOG, independent of study protocol (e.g., home versus laboratory environment; structured versus unstructured protocols) and system design (e.g., one sensor only versus multiple sensors, and one device versus a set of combined devices in different body locations). However, one should be cautious when directly comparing reported performance between studies, for a number of reasons: in particular, one should consider additional factors, such as algorithm used, outcome definitions, data analysis methods, and the intended application of the system.

First, even though FOG is a well-defined symptom [8], what objectively constitutes FOG is unclear. The challenge lies in rigorously defining, from an algorithmic point of view, such a complex event, which can appear in different forms and intensities. Furthermore, the definition of the measured outcome has an important impact upon instrument validity assessment. In this review, some studies only included long-duration FOG episodes. Omitting small FOG episodes may lead to inaccurate estimates of FOG detection rates. A comprehensive definition such as that used by Djuric–Jovici and colleagues [47], differentiating between FOG with trembling and FOG with complete motor blocks prior to video labeling and test properties, seems to address the problem by incorporating different types of FOG events. However, this definition was not used in other studies. A clear and comprehensive definition would improve the comparability of instrument performance.

Second, the intended application of the instrument is another aspect to be considered in FOG detection. It is attractive to aim for rates of 100% specificity and sensitivity. However, this may result in signal processing operations which require substantial computational resources. As illustrated by Ahlrichs [37], the detection of FOG episodes was achieved with high sensitivity and specificity, but the data processing was time-consuming with delays of up to 60 s. Similarly, algorithms with high accuracy may require substantial computational resources which may have an adverse effect on power consumption and hence battery life for non-intrusive, portable devices. This fact may prevent the use of such systems for real-time detection and cueing. Therefore, it is reasonable to conclude that at this point, the acceptability of instrument performance in detection of FOG relate to its application, and many of these algorithms will require substantial mathematical and engineering efforts in order to reduce computational delays to an acceptable level. Furthermore, some algorithms required individual calibration and others did not, which also has practical consequences for applications in clinical and research practice.

Finally, although there exists the potential for these instruments being applied to long-term monitoring in free living conditions, only a few systems were actually validated in the home environment. Therefore, the majority of the technology available lacks “ecological” validation. Thus, further research using larger sample sizes, longer follow-up periods under more realistic home environments is necessary.

### Fall detection and fall risk calculation

Del Din and colleagues described that real-world detection of falls is a substantial challenge from a technical perspective, and almost all evidence in their review was limited to controlled settings and young healthy adults [30]. This finding is confirmed in this review, most clearly illustrated by the fact that we only found one article reporting on fall detection accuracy in PD. However, it is possible that this small number of articles is not only a result of the complexity of capturing falls in PD under realistic, free-living conditions. It certainly highlights an area where the validity of wearable sensors still needs to be examined. In addition, fall risk calculation has the potential to provide objective information before the fall event happens, which may be more valuable than simply counting the number of events and dealing with the consequences.

Fall risk estimation has a clear relevance for clinical practice [58]. Falls are common and disabling, even in early PD [61]. In addition, falls are also related to physical injury [61], high hospitalization cost [62], and social/psychological impact [63], either on their own or due to the anticipatory fear of falling [64]. Even though the number of retrieved articles investigating fall risk calculation was not high, the results seem to confirm the potential for wearable sensors to accurately calculate fall risk for PD.

## Conclusion

This systematic review presents an overview of studies investigating the use of wearable sensors for FOG and falls in Parkinson’s disease. Despite promising validation initiatives, study sample sizes are relatively small, participants are mainly in early stages of the disease, protocols are largely laboratory-based, and there is little consensus on algorithms analysis. Further work in ecological validation, in free-living situations, is necessary. There also is a lack of consistency in outcomes measured, methods of assessing validity, and reported results. Given these limitations, the validation of sensor-derived assessments of PD features would benefit from increased collaboration among researchers, aligning data collection protocols, and sharing data sets.
